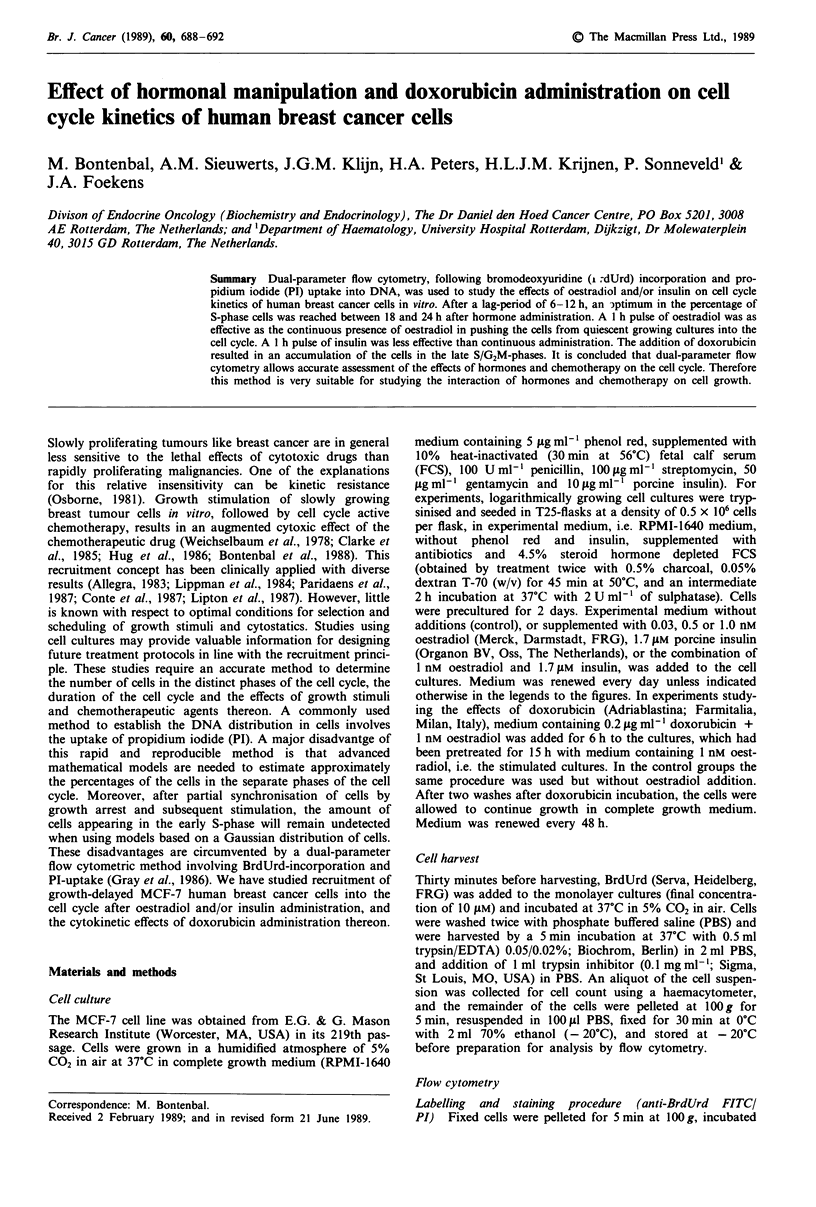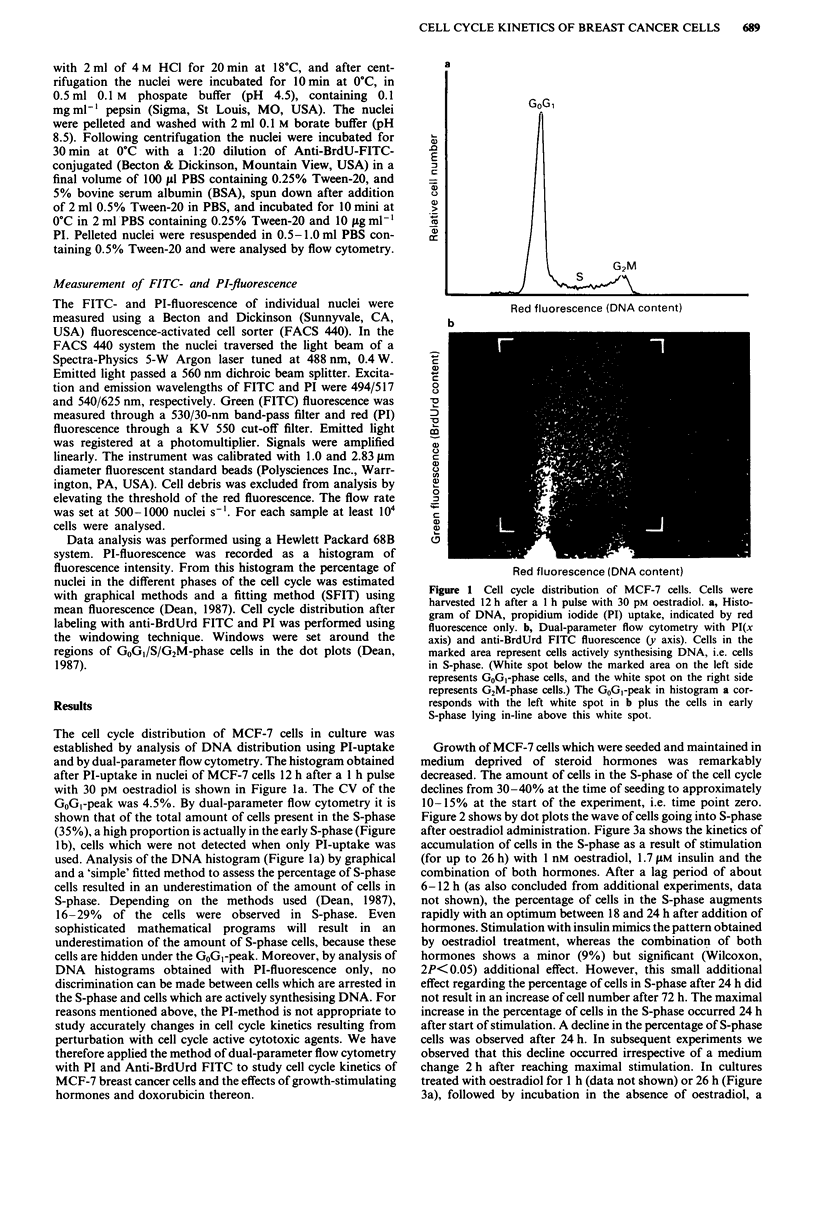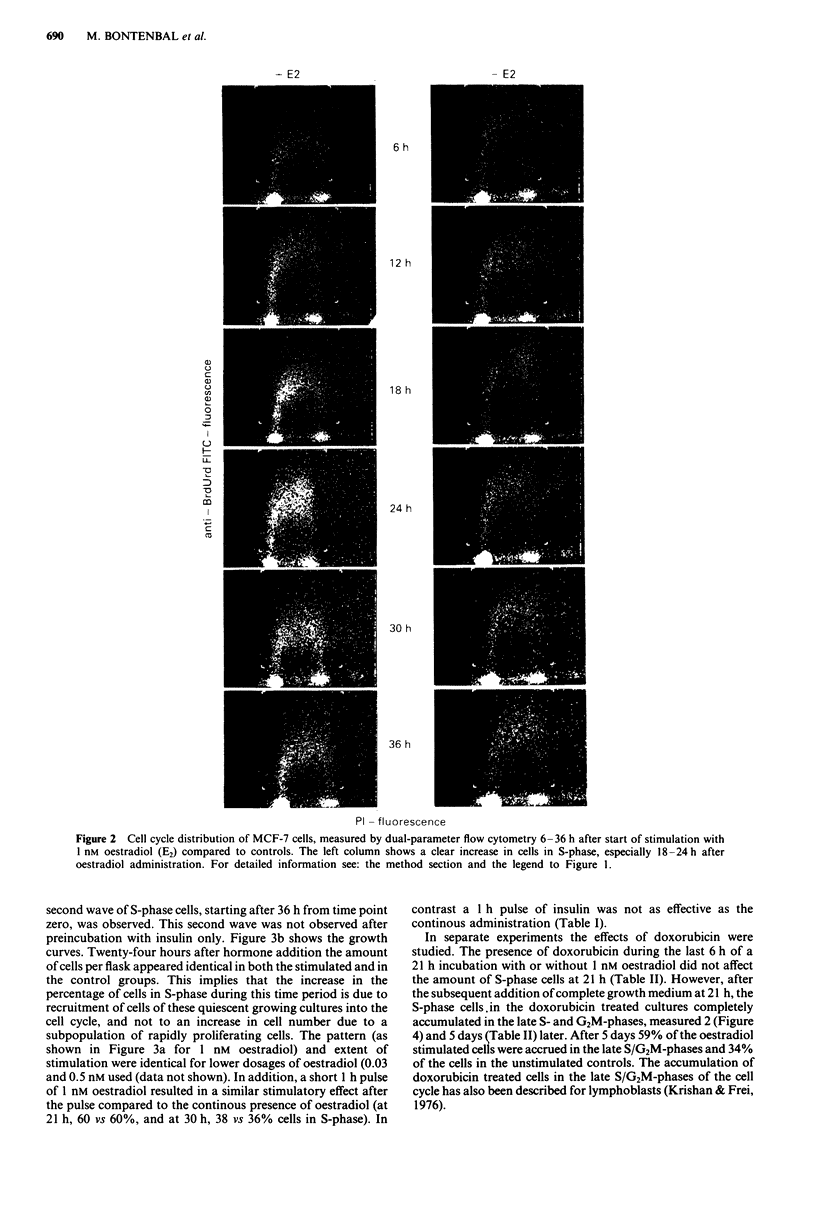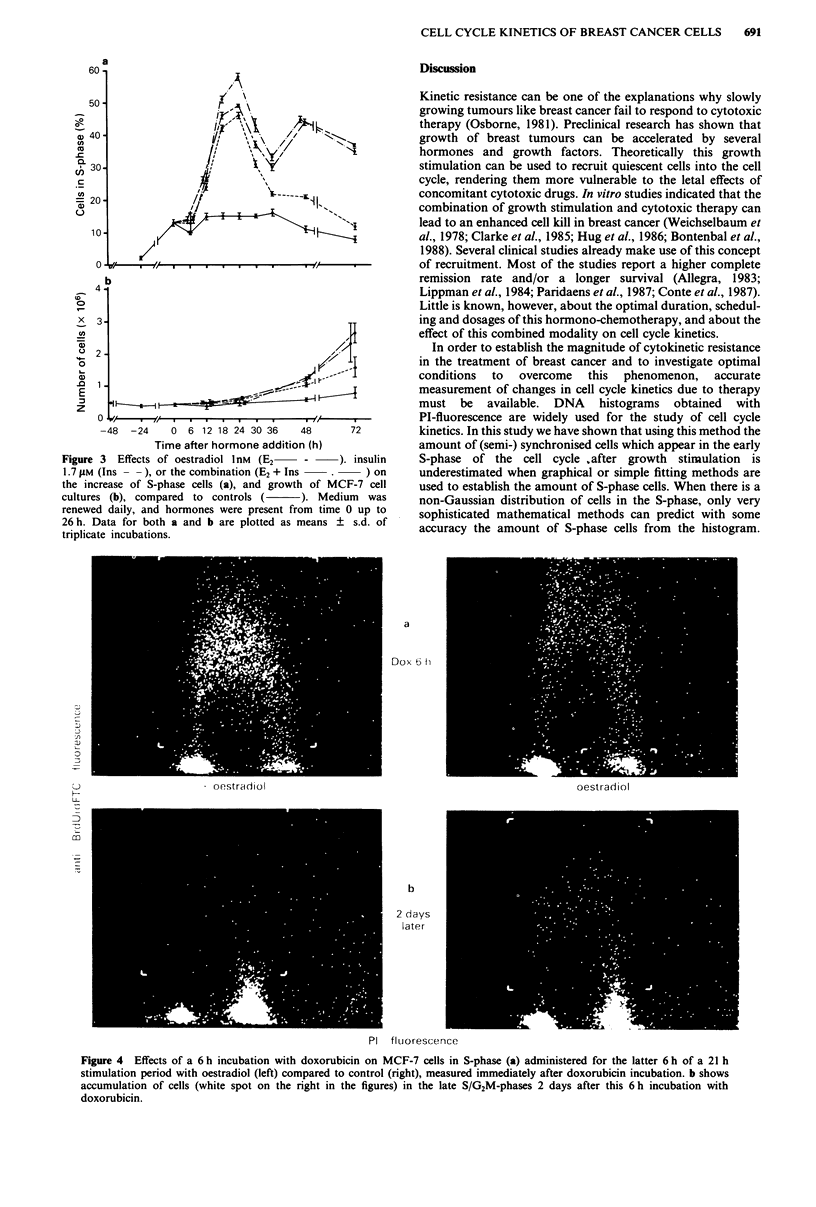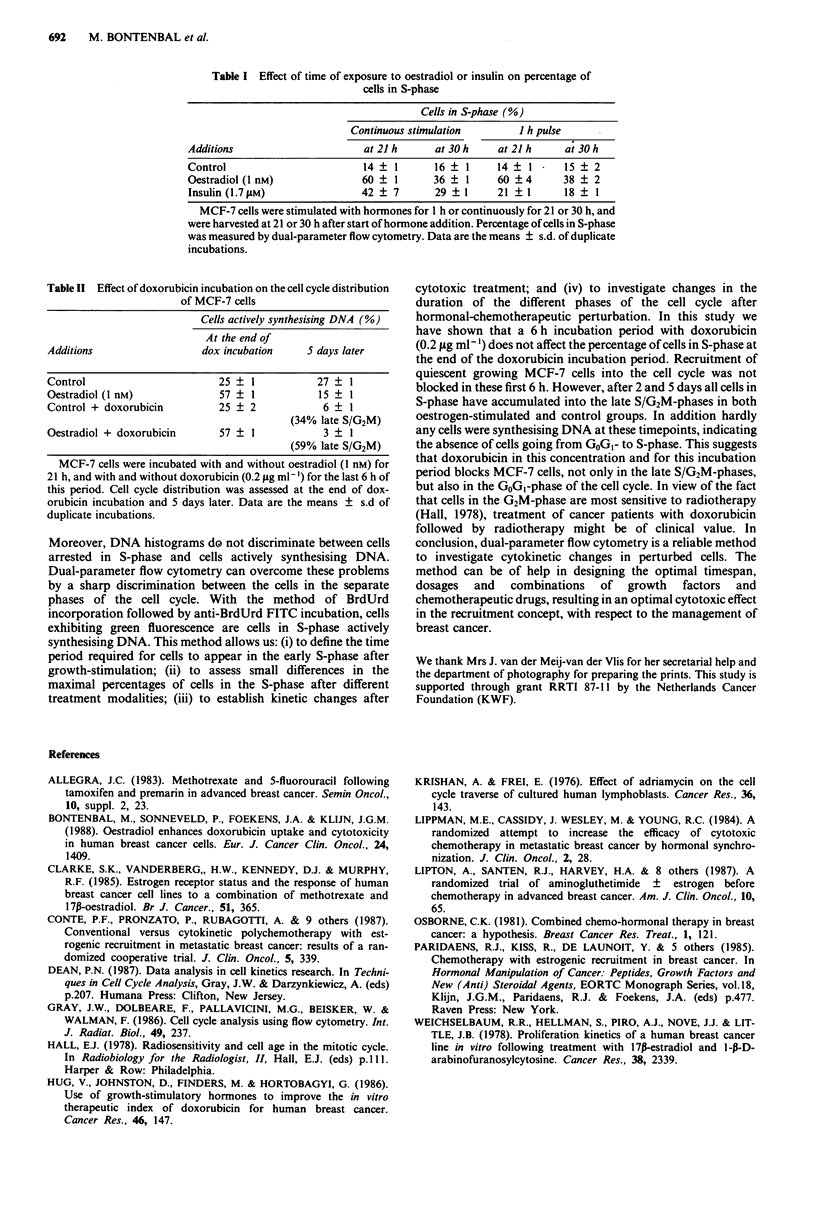# Effect of hormonal manipulation and doxorubicin administration on cell cycle kinetics of human breast cancer cells.

**DOI:** 10.1038/bjc.1989.341

**Published:** 1989-11

**Authors:** M. Bontenbal, A. M. Sieuwerts, J. G. Klijn, H. A. Peters, H. L. Krijnen, P. Sonneveld, J. A. Foekens

**Affiliations:** Division of Endocrine Oncology (Biochemistry and Endocrinology), Dr Daniel den Hoed Cancer Centre, Rotterdam, The Netherlands.

## Abstract

**Images:**


Br. J. Cancer (1989), 60, 688-692 © The Macmillan Press Ltd., 1989

### Effect of hormonal manipulation and doxorubicin administration on cell cycle kinetics of human breast cancer cells

M. Bontenbal, A.M. Sieuwerts, J.G.M. Klijn, H.A. Peters, H.L.J.M. Krijnen, P. Sonneveld[1] & J.A. Foekens

*Divison of Endocrine Oncology (Biochemistry and Endocrinology), The Dr Daniel den Hoed Cancer Centre, PO Box 5201, 3008 AE Rotterdam, The Netherlands; and [1]Department of Haematology, University Hospital Rotterdam, Dijkzigt, Dr Molewaterplein 40, 3015 GD Rotterdam, The Netherlands.*

**Summary** Dual-parameter flow cytometry, following bromodeoxyuridine (BrdUrd) incorporation and propidium iodide (PI) uptake into DNA, was used to study the effects of oestradiol and/or insulin on cell cycle kinetics of human breast cancer cells *in vitro*. After a lag-period of 6-12 h, an optimum in the percentage of S-phase cells was reached between 18 and 24 h after hormone administration. A 1 h pulse of oestradiol was as effective as the continuous presence of oestradiol in pushing the cells from quiescent growing cultures into the cell cycle. A 1 h pulse of insulin was less effective than continuous administration. The addition of doxorubicin resulted in an accumulation of the cells in the late S/$G_2$M-phases. It is concluded that dual-parameter flow cytometry allows accurate assessment of the effects of hormones and chemotherapy on the cell cycle. Therefore this method is very suitable for studying the interaction of hormones and chemotherapy on cell growth.

Slowly proliferating tumours like breast cancer are in general less sensitive to the lethal effects of cytotoxic drugs than rapidly proliferating malignancies. One of the explanations for this relative insensitivity can be kinetic resistance (Osborne, 1981). Growth stimulation of slowly growing breast tumour cells *in vitro*, followed by cell cycle active chemotherapy, results in an augmented cytoxic effect of the chemotherapeutic drug (Weichselbaum *et al*., 1978; Clarke *et al*., 1985; Hug *et al*., 1986; Bontenbal *et al*., 1988). This recruitment concept has been clinically applied with diverse results (Allegra, 1983; Lippman *et al*., 1984; Paridaens *et al*., 1987; Conte *et al*., 1987; Lipton *et al*., 1987). However, little is known with respect to optimal conditions for selection and scheduling of growth stimuli and cytostatics. Studies using cell cultures may provide valuable information for designing future treatment protocols in line with the recruitment principle. These studies require an accurate method to determine the number of cells in the distinct phases of the cell cycle, the duration of the cell cycle and the effects of growth stimuli and chemotherapeutic agents thereon. A commonly used method to establish the DNA distribution in cells involves the uptake of propidium iodide (PI). A major disadvantge of this rapid and reproducible method is that advanced mathematical models are needed to estimate approximately the percentages of the cells in the separate phases of the cell cycle. Moreover, after partial synchronisation of cells by growth arrest and subsequent stimulation, the amount of cells appearing in the early S-phase will remain undetected when using models based on a Gaussian distribution of cells. These disadvantages are circumvented by a dual-parameter flow cytometric method involving BrdUrd-incorporation and PI-uptake (Gray *et al*., 1986). We have studied recruitment of growth-delayed MCF-7 human breast cancer cells into the cell cycle after oestradiol and/or insulin administration, and the cytokinetic effects of doxorubicin administration thereon.

### Materials and methods

### *Cell culture*

The MCF-7 cell line was obtained from E.G. & G. Mason Research Institute (Worcester, MA, USA) in its 219th passage. Cells were grown in a humidified atmosphere of 5% $CO_2$ in air at 37°C in complete growth medium (RPMI-1640 medium containing 5 $\mu g\,ml^{-1}$ phenol red, supplemented with 10% heat-inactivated (30 min at 56°C) fetal calf serum (FCS), 100 U $ml^{-1}$ penicillin, 100 $\mu g\,ml^{-1}$ streptomycin, 50 $\mu g\,ml^{-1}$ gentamycin and 10 $\mu g\,ml^{-1}$ porcine insulin). For experiments, logarithmically growing cell cultures were trypsinised and seeded in T25-flasks at a density of $0.5 \times 10^6$ cells per flask, in experimental medium, i.e. RPMI-1640 medium, without phenol red and insulin, supplemented with antibiotics and 4.5% steroid hormone depleted FCS (obtained by treatment twice with 0.5% charcoal, 0.05% dextran T-70 (w/v) for 45 min at 50°C, and an intermediate 2 h incubation at 37°C with 2 U $ml^{-1}$ of sulphatase). Cells were precultured for 2 days. Experimental medium without additions (control), or supplemented with 0.03, 0.5 or 1.0 nM oestradiol (Merck, Darmstadt, FRG), 1.7 $\mu$M porcine insulin (Organon BV, Oss, The Netherlands), or the combination of 1 nM oestradiol and 1.7 $\mu$M insulin, was added to the cell cultures. Medium was renewed every day unless indicated otherwise in the legends to the figures. In experiments studying the effects of doxorubicin (Adriablastina; Farmitalia, Milan, Italy), medium containing 0.2 $\mu g\,ml^{-1}$ doxorubicin + 1 nM oestradiol was added for 6 h to the cultures, which had been pretreated for 15 h with medium containing 1 nM oestradiol, i.e. the stimulated cultures. In the control groups the same procedure was used but without oestradiol addition. After two washes after doxorubicin incubation, the cells were allowed to continue growth in complete growth medium. Medium was renewed every 48 h.

### *Cell harvest*

Thirty minutes before harvesting, BrdUrd (Serva, Heidelberg, FRG) was added to the monolayer cultures (final concentration of 10 $\mu$M) and incubated at 37°C in 5% $CO_2$ in air. Cells were washed twice with phosphate buffered saline (PBS) and were harvested by a 5 min incubation at 37°C with 0.5 ml trypsin/EDTA) 0.05/0.02%; Biochrom, Berlin) in 2 ml PBS, and addition of 1 ml trypsin inhibitor (0.1 mg $ml^{-1}$; Sigma, St Louis, MO, USA) in PBS. An aliquot of the cell suspension was collected for cell count using a haemacytometer, and the remainder of the cells were pelleted at 100 *g* for 5 min, resuspended in 100 $\mu$l PBS, fixed for 30 min at 0°C with 2 ml 70% ethanol (– 20°C), and stored at – 20°C before preparation for analysis by flow cytometry.

### *Flow cytometry*

*Labelling and staining procedure (anti-BrdUrd FITC/PI)* Fixed cells were pelleted for 5 min at 100 *g*, incubated

Correspondence: M. Bontenbal.
Received 2 February 1989; and in revised form 21 June 1989.

with 2 ml of 4 M HCl for 20 min at 18°C, and after centrifugation the nuclei were incubated for 10 min at 0°C, in 0.5 ml 0.1 M phospate buffer (pH 4.5), containing 0.1 mg $ml^{-1}$ pepsin (Sigma, St Louis, MO, USA). The nuclei were pelleted and washed with 2 ml 0.1 M borate buffer (pH 8.5). Following centrifugation the nuclei were incubated for 30 min at 0°C with a 1:20 dilution of Anti-BrdU-FITC-conjugated (Becton & Dickinson, Mountain View, USA) in a final volume of 100 µl PBS containing 0.25% Tween-20, and 5% bovine serum albumin (BSA), spun down after addition of 2 ml 0.5% Tween-20 in PBS, and incubated for 10 mini at 0°C in 2 ml PBS containing 0.25% Tween-20 and 10 µg $ml^{-1}$ PI. Pelleted nuclei were resuspended in 0.5–1.0 ml PBS containing 0.5% Tween-20 and were analysed by flow cytometry.

*Measurement of FITC- and PI-fluorescence*

The FITC- and PI-fluorescence of individual nuclei were measured using a Becton and Dickinson (Sunnyvale, CA, USA) fluorescence-activated cell sorter (FACS 440). In the FACS 440 system the nuclei traversed the light beam of a Spectra-Physics 5-W Argon laser tuned at 488 nm, 0.4 W. Emitted light passed a 560 nm dichroic beam splitter. Excitation and emission wavelengths of FITC and PI were 494/517 and 540/625 nm, respectively. Green (FITC) fluorescence was measured through a 530/30-nm band-pass filter and red (PI) fluorescence through a KV 550 cut-off filter. Emitted light was registered at a photomultiplier. Signals were amplified linearly. The instrument was calibrated with 1.0 and 2.83 µm diameter fluorescent standard beads (Polysciences Inc., Warrington, PA, USA). Cell debris was excluded from analysis by elevating the threshold of the red fluorescence. The flow rate was set at 500–1000 nuclei $s^{-1}$. For each sample at least $10^4$ cells were analysed.

Data analysis was performed using a Hewlett Packard 68B system. PI-fluorescence was recorded as a histogram of fluorescence intensity. From this histogram the percentage of nuclei in the different phases of the cell cycle was estimated with graphical methods and a fitting method (SFIT) using mean fluorescence (Dean, 1987). Cell cycle distribution after labeling with anti-BrdUrd FITC and PI was performed using the windowing technique. Windows were set around the regions of $G_0G_1$/S/$G_2M$-phase cells in the dot plots (Dean, 1987).

### Results

The cell cycle distribution of MCF-7 cells in culture was established by analysis of DNA distribution using PI-uptake and by dual-parameter flow cytometry. The histogram obtained after PI-uptake in nuclei of MCF-7 cells 12 h after a 1 h pulse with 30 pM oestradiol is shown in Figure 1a. The CV of the $G_0G_1$-peak was 4.5%. By dual-parameter flow cytometry it is shown that of the total amount of cells present in the S-phase (35%), a high proportion is actually in the early S-phase (Figure 1b), cells which were not detected when only PI-uptake was used. Analysis of the DNA histogram (Figure 1a) by graphical and a 'simple' fitted method to assess the percentage of S-phase cells resulted in an underestimation of the amount of cells in S-phase. Depending on the methods used (Dean, 1987), 16–29% of the cells were observed in S-phase. Even sophisticated mathematical programs will result in an underestimation of the amount of S-phase cells, because these cells are hidden under the $G_0G_1$-peak. Moreover, by analysis of DNA histograms obtained with PI-fluorescence only, no discrimination can be made between cells which are arrested in the S-phase and cells which are actively synthesising DNA. For reasons mentioned above, the PI-method is not appropriate to study accurately changes in cell cycle kinetics resulting from perturbation with cell cycle active cytotoxic agents. We have therefore applied the method of dual-parameter flow cytometry with PI and Anti-BrdUrd FITC to study cell cycle kinetics of MCF-7 breast cancer cells and the effects of growth-stimulating hormones and doxorubicin thereon.

**Figure 1** Cell cycle distribution of MCF-7 cells. Cells were harvested 12 h after a 1 h pulse with 30 pM oestradiol. **a**, Histogram of DNA, propidium iodide (PI) uptake, indicated by red fluorescence only. **b**, Dual-parameter flow cytometry with PI($x$ axis) and anti-BrdUrd FITC fluorescence ($y$ axis). Cells in the marked area represent cells actively synthesising DNA, i.e. cells in S-phase. (White spot below the marked area on the left side represents $G_0G_1$-phase cells, and the white spot on the right side represents $G_2M$-phase cells.) The $G_0G_1$-peak in histogram **a** corresponds with the left white spot in **b** plus the cells in early S-phase lying in-line above this white spot.

Growth of MCF-7 cells which were seeded and maintained in medium deprived of steroid hormones was remarkably decreased. The amount of cells in the S-phase of the cell cycle declines from 30–40% at the time of seeding to approximately 10–15% at the start of the experiment, i.e. time point zero. Figure 2 shows by dot plots the wave of cells going into S-phase after oestradiol administration. Figure 3a shows the kinetics of accumulation of cells in the S-phase as a result of stimulation (for up to 26 h) with 1 nM oestradiol, 1.7 µM insulin and the combination of both hormones. After a lag period of about 6–12 h (as also concluded from additional experiments, data not shown), the percentage of cells in the S-phase augments rapidly with an optimum between 18 and 24 h after addition of hormones. Stimulation with insulin mimics the pattern obtained by oestradiol treatment, whereas the combination of both hormones shows a minor (9%) but significant (Wilcoxon, $2P < 0.05$) additional effect. However, this small additional effect regarding the percentage of cells in S-phase after 24 h did not result in an increase of cell number after 72 h. The maximal increase in the percentage of cells in the S-phase occurred 24 h after start of stimulation. A decline in the percentage of S-phase cells was observed after 24 h. In subsequent experiments we observed that this decline occurred irrespective of a medium change 2 h after reaching maximal stimulation. In cultures treated with oestradiol for 1 h (data not shown) or 26 h (Figure 3a), followed by incubation in the absence of oestradiol, a

**Figure 2** Cell cycle distribution of MCF-7 cells, measured by dual-parameter flow cytometry 6–36 h after start of stimulation with 1 nM oestradiol ($E_2$) compared to controls. The left column shows a clear increase in cells in S-phase, especially 18–24 h after oestradiol administration. For detailed information see: the method section and the legend to Figure 1.

second wave of S-phase cells, starting after 36 h from time point zero, was observed. This second wave was not observed after preincubation with insulin only. Figure 3b shows the growth curves. Twenty-four hours after hormone addition the amount of cells per flask appeared identical in both the stimulated and in the control groups. This implies that the increase in the percentage of cells in S-phase during this time period is due to recruitment of cells of these quiescent growing cultures into the cell cycle, and not to an increase in cell number due to a subpopulation of rapidly proliferating cells. The pattern (as shown in Figure 3a for 1 nM oestradiol) and extent of stimulation were identical for lower dosages of oestradiol (0.03 and 0.5 nM used (data not shown). In addition, a short 1 h pulse of 1 nM oestradiol resulted in a similar stimulatory effect after the pulse compared to the continous presence of oestradiol (at 21 h, 60 *vs* 60%, and at 30 h, 38 *vs* 36% cells in S-phase). In contrast a 1 h pulse of insulin was not as effective as the continous administration (Table I).

In separate experiments the effects of doxorubicin were studied. The presence of doxorubicin during the last 6 h of a 21 h incubation with or without 1 nM oestradiol did not affect the amount of S-phase cells at 21 h (Table II). However, after the subsequent addition of complete growth medium at 21 h, the S-phase cells.in the doxorubicin treated cultures completely accumulated in the late S- and $G_2M$-phases, measured 2 (Figure 4) and 5 days (Table II) later. After 5 days 59% of the oestradiol stimulated cells were accrued in the late S/$G_2M$-phases and 34% of the cells in the unstimulated controls. The accumulation of doxorubicin treated cells in the late S/$G_2M$-phases of the cell cycle has also been described for lymphoblasts (Krishan & Frei, 1976).

**Figure 3** Effects of oestradiol 1nM ($E_2$—— - ——). insulin 1.7 μM (Ins – – ), or the combination ($E_2$ + Ins —— . —— ) on the increase of S-phase cells (**a**), and growth of MCF-7 cell cultures (**b**), compared to controls (———). Medium was renewed daily, and hormones were present from time 0 up to 26 h. Data for both **a** and **b** are plotted as means ± s.d. of triplicate incubations.

### Discussion

Kinetic resistance can be one of the explanations why slowly growing tumours like breast cancer fail to respond to cytotoxic therapy (Osborne, 1981). Preclinical research has shown that growth of breast tumours can be accelerated by several hormones and growth factors. Theoretically this growth stimulation can be used to recruit quiescent cells into the cell cycle, rendering them more vulnerable to the letal effects of concomitant cytotoxic drugs. *In vitro* studies indicated that the combination of growth stimulation and cytotoxic therapy can lead to an enhanced cell kill in breast cancer (Weichselbaum *et al.*, 1978; Clarke *et al.*, 1985; Hug *et al.*, 1986; Bontenbal *et al.*, 1988). Several clinical studies already make use of this concept of recruitment. Most of the studies report a higher complete remission rate and/or a longer survival (Allegra, 1983; Lippman *et al.*, 1984; Paridaens *et al.*, 1987; Conte *et al.*, 1987). Little is known, however, about the optimal duration, scheduling and dosages of this hormono-chemotherapy, and about the effect of this combined modality on cell cycle kinetics.

In order to establish the magnitude of cytokinetic resistance in the treatment of breast cancer and to investigate optimal conditions to overcome this phenomenon, accurate measurement of changes in cell cycle kinetics due to therapy must be available. DNA histograms obtained with PI-fluorescence are widely used for the study of cell cycle kinetics. In this study we have shown that using this method the amount of (semi-) synchronised cells which appear in the early S-phase of the cell cycle ,after growth stimulation is underestimated when graphical or simple fitting methods are used to establish the amount of S-phase cells. When there is a non-Gaussian distribution of cells in the S-phase, only very sophisticated mathematical methods can predict with some accuracy the amount of S-phase cells from the histogram.

**Figure 4** Effects of a 6 h incubation with doxorubicin on MCF-7 cells in S-phase (**a**) administered for the latter 6 h of a 21 h stimulation period with oestradiol (left) compared to control (right), measured immediately after doxorubicin incubation. **b** shows accumulation of cells (white spot on the right in the figures) in the late $S/G_2M$-phases 2 days after this 6 h incubation with doxorubicin.

**Table I** Effect of time of exposure to oestradiol or insulin on percentage of cells in S-phase

| | *Cells in S-phase (%)* | | | |
|---|---|---|---|---|
| | *Continuous stimulation* | | *1 h pulse* | |
| *Additions* | *at 21 h* | *at 30 h* | *at 21 h* | *at 30 h* |
| Control | 14 ± 1 | 16 ± 1 | 14 ± 1 | 15 ± 2 |
| Oestradiol (1 nM) | 60 ± 1 | 36 ± 1 | 60 ± 4 | 38 ± 2 |
| Insulin (1.7 μM) | 42 ± 7 | 29 ± 1 | 21 ± 1 | 18 ± 1 |

MCF-7 cells were stimulated with hormones for 1 h or continuously for 21 or 30 h, and were harvested at 21 or 30 h after start of hormone addition. Percentage of cells in S-phase was measured by dual-parameter flow cytometry. Data are the means ± s.d. of duplicate incubations.

**Table II** Effect of doxorubicin incubation on the cell cycle distribution of MCF-7 cells

| | *Cells actively synthesising DNA (%)* | |
|---|---|---|
| *Additions* | *At the end of dox incubation* | *5 days later* |
| Control | 25 ± 1 | 27 ± 1 |
| Oestradiol (1 nM) | 57 ± 1 | 15 ± 1 |
| Control + doxorubicin | 25 ± 2 | 6 ± 1 (34% late $S/G_2M$) |
| Oestradiol + doxorubicin | 57 ± 1 | 3 ± 1 (59% late $S/G_2M$) |

MCF-7 cells were incubated with and without oestradiol (1 nM) for 21 h, and with and without doxorubicin (0.2 μg ml$^{-1}$) for the last 6 h of this period. Cell cycle distribution was assessed at the end of doxorubicin incubation and 5 days later. Data are the means ± s.d of duplicate incubations.

Moreover, DNA histograms do not discriminate between cells arrested in S-phase and cells actively synthesising DNA. Dual-parameter flow cytometry can overcome these problems by a sharp discrimination between the cells in the separate phases of the cell cycle. With the method of BrdUrd incorporation followed by anti-BrdUrd FITC incubation, cells exhibiting green fluorescence are cells in S-phase actively synthesising DNA. This method allows us: (i) to define the time period required for cells to appear in the early S-phase after growth-stimulation; (ii) to assess small differences in the maximal percentages of cells in the S-phase after different treatment modalities; (iii) to establish kinetic changes after cytotoxic treatment; and (iv) to investigate changes in the duration of the different phases of the cell cycle after hormonal-chemotherapeutic perturbation. In this study we have shown that a 6 h incubation period with doxorubicin (0.2 μg ml$^{-1}$) does not affect the percentage of cells in S-phase at the end of the doxorubicin incubation period. Recruitment of quiescent growing MCF-7 cells into the cell cycle was not blocked in these first 6 h. However, after 2 and 5 days all cells in S-phase have accumulated into the late $S/G_2M$-phases in both oestrogen-stimulated and control groups. In addition hardly any cells were synthesising DNA at these timepoints, indicating the absence of cells going from $G_0G_1$- to S-phase. This suggests that doxorubicin in this concentration and for this incubation period blocks MCF-7 cells, not only in the late $S/G_2M$-phases, but also in the $G_0G_1$-phase of the cell cycle. In view of the fact that cells in the $G_2M$-phase are most sensitive to radiotherapy (Hall, 1978), treatment of cancer patients with doxorubicin followed by radiotherapy might be of clinical value. In conclusion, dual-parameter flow cytometry is a reliable method to investigate cytokinetic changes in perturbed cells. The method can be of help in designing the optimal timespan, dosages and combinations of growth factors and chemotherapeutic drugs, resulting in an optimal cytotoxic effect in the recruitment concept, with respect to the management of breast cancer.

We thank Mrs J. van der Meij-van der Vlis for her secretarial help and the department of photography for preparing the prints. This study is supported through grant RRTI 87-11 by the Netherlands Cancer Foundation (KWF).

### References

ALLEGRA, J.C. (1983). Methotrexate and 5-fluorouracil following tamoxifen and premarin in advanced breast cancer. *Semin Oncol.*, **10,** suppl. 2, 23.

BONTENBAL, M., SONNEVELD, P., FOEKENS, J.A. & KLIJN, J.G.M. (1988). Oestradiol enhances doxorubicin uptake and cytotoxicity in human breast cancer cells. *Eur. J. Cancer Clin. Oncol.*, **24,** 1409.

CLARKE, S.K., VANDERBERG,, H.W., KENNEDY, D.J. & MURPHY, R.F. (1985). Estrogen receptor status and the response of human breast cancer cell lines to a combination of methotrexate and 17β-oestradiol. *Br J. Cancer.*, **51,** 365.

CONTE, P.F., PRONZATO, P., RUBAGOTTI, A. & 9 others (1987). Conventional versus cytokinetic polychemotherapy with estrogenic recruitment in metastatic breast cancer: results of a randomized cooperative trial. *J. Clin. Oncol.*, **5,** 339.

DEAN, P.N. (1987). Data analysis in cell kinetics research. In *Techniques in Cell Cycle Analysis*, Gray, J.W. & Darzynkiewicz, A. (eds) p.207. Humana Press: Clifton, New Jersey.

GRAY, J.W., DOLBEARE, F., PALLAVICINI, M.G., BEISKER, W. & WALMAN, F. (1986). Cell cycle analysis using flow cytometry. *Int. J. Radiat. Biol.*, **49,** 237.

HALL, E.J. (1978). Radiosensitivity and cell age in the mitotic cycle. In *Radiobiology for the Radiologist, II*, Hall, E.J. (eds) p.111. Harper & Row: Philadelphia.

HUG, V., JOHNSTON, D., FINDERS, M. & HORTOBAGYI, G. (1986). Use of growth-stimulatory hormones to improve the *in vitro* therapeutic index of doxorubicin for human breast cancer. *Cancer Res.*, **46,** 147.

KRISHAN, A. & FREI, E. (1976). Effect of adriamycin on the cell cycle traverse of cultured human lymphoblasts. *Cancer Res.*, **36,** 143.

LIPPMAN, M.E., CASSIDY, J. WESLEY, M. & YOUNG, R.C. (1984). A randomized attempt to increase the efficacy of cytotoxic chemotherapy in metastatic breast cancer by hormonal synchronization. *J. Clin. Oncol.*, **2,** 28.

LIPTON, A., SANTEN, R.J., HARVEY, H.A. & 8 others (1987). A randomized trial of aminogluthetimide ± estrogen before chemotherapy in advanced breast cancer. *Am. J. Clin. Oncol.*, **10,** 65.

OSBORNE, C.K. (1981). Combined chemo-hormonal therapy in breast cancer: a hypothesis. *Breast Cancer Res. Treat.*, **1,** 121.

PARIDAENS, R.J., KISS, R., DE LAUNOIT, Y. & 5 others (1985). Chemotherapy with estrogenic recruitment in breast cancer. In *Hormonal Manipulation of Cancer: Peptides, Growth Factors and New (Anti) Steroidal Agents*, EORTC Monograph Series, vol.18, Klijn, J.G.M., Paridaens, R.J. & Foekens, J.A. (eds) p.477. Raven Press: New York.

WEICHSELBAUM, R.R., HELLMAN, S., PIRO, A.J., NOVE, J.J. & LITTLE, J.B. (1978). Proliferation kinetics of a human breast cancer line *in vitro* following treatment with 17β-estradiol and 1-β-D-arabinofuranosylcytosine. *Cancer Res.*, **38,** 2339.